# Listeriolysin O Affects the Permeability of Caco-2 Monolayer in a Pore-Dependent and Ca^2+^-Independent Manner

**DOI:** 10.1371/journal.pone.0130471

**Published:** 2015-06-18

**Authors:** Miša Mojca Cajnko, Maja Marušić, Matic Kisovec, Nejc Rojko, Mojca Benčina, Simon Caserman, Gregor Anderluh

**Affiliations:** 1 Laboratory for Molecular Biology and Nanobiotechnology, National Institute of Chemistry, Hajdrihova 19, 1000, Ljubljana, Slovenia; 2 Laboratory for Biotechnology, National Institute of Chemistry, Hajdrihova 19, 1000, Ljubljana, Slovenia; 3 Department of Biology, Biotechnical Faculty, University of Ljubljana, Jamnikarjeva 101, 1000, Ljubljana, Slovenia; CEA (Atomic and alternative energies commission), FRANCE

## Abstract

*Listeria monocytogenes* is a food and soil-borne pathogen that secretes a pore-forming toxin listeriolysin O (LLO) as its major virulence factor. We tested the effects of LLO on an intestinal epithelial cell line Caco-2 and compared them to an unrelated pore-forming toxin equinatoxin II (EqtII). Results showed that apical application of both toxins causes a significant drop in transepithelial electrical resistance (TEER), with higher LLO concentrations or prolonged exposure time needed to achieve the same magnitude of response than with EqtII. The drop in TEER was due to pore formation and coincided with rearrangement of claudin-1 within tight junctions and associated actin cytoskeleton; however, no significant increase in permeability to fluorescein or 3 kDa FITC-dextran was observed. Influx of calcium after pore formation affected the magnitude of the drop in TEER. Both toxins exhibit similar effects on epithelium morphology and physiology. Importantly, LLO action upon the membrane is much slower and results in compromised epithelium on a longer time scale at lower concentrations than EqtII. This could favor listerial invasion in hosts resistant to E-cadherin related infection.

## Introduction

*Listeria monocytogenes* is an invasive Gram-positive enteric pathogen. It can cause life-threatening diseases like septicemia, meningitis, meningoencephalitis, and severe gastroenteritis [1]. The bacterium expresses multiple virulence factors that help its entry and survival in host cells [2–4]. One of its major virulence factors is a pore-forming toxin listeriolysin O (LLO) [5]. LLO belongs to a family of cholesterol-dependent cytolysins (CDCs), pore-forming toxins produced by many Gram-positive bacteria [6]. CDCs are 50–70 kDa proteins that bind to cholesterol in host cell membranes and form large pores comprised of 35–50 monomers with a diameter of 25–40 nm [7]. To invade non-phagocytic cells at least two listerial surface proteins are usually employed, internalin A (InlA), which binds to E-cadherin, and internalin B (InlB), which binds to the extracellular domain of c-Met tyrosine kinase [8–11]. E-cadherin is expressed by intestinal epithelial cells and binding of InlA to E-cadherin is essential for the translocation of *L*. *monocytogenes* across the intestinal barrier via transcytosis [12,13]. InlA-E-cadherin interaction takes place at multicellular junction sites where single intestinal senescent cells are expelled from the epithelium by extrusion [14].

Alternatively, the bacterium can invade non-phagocytic cells with a help of LLO. Calcium influx triggered by LLO pore-formation on the plasma membrane induces internalization of the bacterium into the primary vacuole [15,16]. Furthermore, LLO, together with the phospholipase C, another important virulence factor of *L*. *monocytogenes* [17,18], is essential for the disruption of the primary vacuole and release of the bacterium into the cytosol [19]. Once inside the cytosol the bacterium recruits the host’s F-actin polimerization machinery [20,21] to propel itself until it forms an extracellular protrusion, which enables it to invade neighboring cells [22,23]. Once the protrusion is engulfed by the adjacent cell, it forms a secondary vacuole with two membranes and *L*. *monocytogenes* trapped inside. LLO is then again required for the disruption of the secondary vacuole and release of bacterium into the cytosol [24]. Many studies have shown, however, that the role of LLO is more complex. It has been shown that LLO released into the extracellular space and its binding to the target cell membrane may induce apoptosis [25], endocytosis [16], histone modifications [26], phosphorylation of certain MAP kinases [27], cytokine expression [28–31], chloride secretion [32], release of calcium from intracellular stores [33], trigger a cell quiescent-like state [27], activate caspase-7 [34] and unfolded-protein response [35], impair SUMOylation [36], disrupt mitochondrial dynamics [37] and impair barrier function [32].

The aim of this work was to elucidate Caco-2 cell monolayer response after exposure to LLO. The well-established InlA–E-cadherin route of listeria invasion is not available in one of the most efficient vectors like mice or rats [38]. Therefore, our hypothesis was that LLO action upon apical membrane of enterocytes could serve as the initial trigger of response reducing barrier integrity through pore formation. Living bacteria in feces [39] indicate that *L*. *monocytogenes* is found all throughout the gastrointestinal tract. Compromising of barrier function could thus facilitate bacterial entry into enterocytes as well as spread through the mucosa into the rest of the body. Therefore, we examined the effects of LLO on the integrity of the Caco-2 epithelium. Caco-2 cell line is derived from a colorectal adenocarcinoma and exhibits several morphological and biochemical characteristics of small intestinal enterocytes and is often used for permeability studies of various molecules [40–42]. For comparison we used Equinatoxin II (EqtII), a pore-forming toxin from the sea anemone *Actinia equina*, in order to distinguish between effects due to pore-formation or any other LLO-specific effects on Caco-2 cells. EqtII belongs to pore-forming toxin family of actinoporins that forms pores by a distinctively different mechanism than LLO [43–45]. Actinoporin pores are much smaller in size (2 nm in comparison to 25–40 nm of CDC pores) [46–48]. In addition, actinoporins are targeting sphingomyelin, a lipid abundantly present in the outer lipid layer of plasma membrane [49], while LLO requires significant concentrations of cholesterol for efficient lipid membrane binding [50]. Here we show that both toxins cause a fast and profound drop in transepithelial electrical resistance (TEER) of the Caco-2 cell monolayer due to pore formation and trigger changes in tight junction complex as well as actin cytoskeleton. We also show that effect of calcium influx through pores or its release from intracellular stores is rather limited during this process and that the increased apical membrane permeability to potassium has no notable effect on TEER. Our results contribute to a better understanding of cellular responses to pore-forming proteins and pathogenicity mechanism of *L*. *monocytogenes*.

## Materials and Methods

### Materials

Caco-2 cell line (American Tissue Culture Collection), Minimum essential medium eagle (MEM), Dulbecco's modified eagle's medium (DMEM), trypsin, heat inactivated fetal bovine serum (HFBS), non-essential amino acids, bovine serum albumin (BSA), dimethyl sulfoxide (DMSO), 3-(4,5-dimethylthiazol-2-yl)-2,5-diphenyltetrazolium bromide (MTT), fluorescein sodium, 2-((2-(Bis(carboxymethyl)amino)ethyl)(carboxymethyl)amino)acetic acid (EDTA), ethylene glycol-bis(2-aminoethylether)-*N*, *N*, *N′*, *N′*-tetraacetic acid (EGTA), ionomycin calcium salt, nigericin sodium salt from *Streptomyces hygroscopicus*, Tween 20, chloroform, 2-(N-morpholino)ethanesulfonic acid (MES), sodium chloride (all from Sigma Aldrich), SYTOX green nucleic acid stain, Alexa fluor 488 phalloidin, DAPI, rabbit-anti-claudin-1 primary antibodies, mouse-anti-occludin primary antibodies, mouse-anti-E-cadherin primary antibodies, goat-anti-mouse AlexaFluor488 secondary antibodies, goat-anti-rabbit AlexaFluor488 secondary antibodies, FITC-dextran 3 (all from Invitrogen molecular probes), methanol, acetone, ethanol, 4% paraformaldehyde (all from Merck), antibiotic/antimycotic (Gibco), L-glutamine (PAA), lactate dehydrogenase (LDH) substrate (Promega), dithiothreitol (DTT) (Gold Biotechnology), goat-anti-rabbit HRP (Abcam), Tris(hydroxymethyl)-aminomethane (Tris) (Merck), Triton X-100 (Fluca), 1-palmitoyl-2-oleoyl-sn-glycero-3-phosphocholine (POPC), cholesterol (Avanti).

LLO and its double cysteine mutant LLO^A318C-L334C^ were produced in *E*. *coli* BL21(DE3)pLysS strain using pPROEX-HTb vector and standard molecular biology approaches essentially as described in [51]. Recombinant proteins were purified by Ni-NTA chromatography. Briefly, cells were pelleted after the fermentation and resuspended in the lysis buffer (50 mM NaH_2_PO_4_/Na_2_HPO_4_, 250 mM NaCl, 10% (v:v) glycerol, pH 6.5) and stored at -80°C. The next day cells were thawed and β-mercaptoethanol and phenylmethanesulfonylfluoride were added to the final concentration of 5 mM and 2 mM, respectively. Cells were sonicated on ice and then centrifuged at 44 000 *g* for 50 min at 4°C. The supernatant was loaded on Ni-NTA resin (Qiagen), and the hexa-histidine-tagged protein was eluted with 50 mM NaH_2_PO_4_/Na_2_HPO_4_, 300 mM NaCl, 300 mM imidazole, 5% (v:v) glycerol, pH 6.5. Tobacco etch virus protease was added to the eluted protein and the whole mixture was dialyzed against 20 mM Tris-HCl, 200 mM NaCl, 5% (v:v) glycerol, pH 7 at 4°C overnight. The uncleaved protein and protease were removed by passing the sample over Ni-NTA column. The unbound fraction represented the cleaved LLO or mutant. All final protein concentrations were determined spectrophotometrically by measuring absorbance at 280 nm. Extinction coefficients were derived from primary sequences by using ExPASy tools (http://www.expasy.org/). All purified proteins were aliquoted and stored at -80°C. EqtII and its disulfide mutant EqtII^K8C-V69C^ were prepared and assayed for activity as described previously [52,53].

### Cell cultures

Caco-2 cells were grown at 37°C in the growth medium MEM supplemented with 10% (v:v) fetal bovine serum, 1% L-glutamine and 1% non-essential amino acids, under the atmosphere containing 5% CO_2_. Cells were subcultured every 7 days or after reaching 80–90% confluence. Caco-2 cells were then seeded on transwell permeable supports (Corning), 96-well microtiter plates (Corning) or chamber slides (Ibidi) and cultured for 1 or 3 weeks depending on the experiment. The test medium used was DMEM supplemented with 1% glutamine, 1% antibiotic-antimycotic and 10% FBS. The test medium was changed every second or third day.

### Transepithelial electrical resistance measurements (TEER)

For measurements of TEER 200 000 cells/ml were seeded on transwell permeable supports and cultured for 3 weeks. TEER measurements were done using Millipore Millicell ERS probe MERSSTX01 and ERS-2 Epithelial volt-ohm meter. Prior to every experiment cells were washed in serum-free medium by exchanging the medium in both compartments with DMEM. Different concentrations of LLO, EqtII, or mutants were added to the apical or basolateral compartment and TEER was measured for 3 min. Mutant proteins were incubated either with the reducing (1 mM dithiotreitol) or oxidizing (0.5 mM phenanthroline, 0.1 mM CuSO_4_) agent for 20 min at room temperature prior to addition to Caco-2 cells.

When regeneration of Caco-2 monolayer was monitored, cells were treated with various concentrations of LLO or EqtII and TEER was measured for the first 3 min, then every hour for 7 hours and again after 24 hours. During the experiment, three hours after the initial toxin treatment, cells were rinsed by the fresh test medium and kept in it for the rest of the regeneration period.

To assess effects of calcium, Caco-2 cells were first washed with DMEM and then with either phosphate buffered saline (PBS) or DMEM with 2.5 mM EDTA or EGTA and treated with 125 nM LLO or 100 nM EqtII in the same buffer or medium. For intra- and extra-cellular calcium chelation cells were incubated with 50 μM BAPTA-AM in DMEM for 45 min at 37°C, and then washed with PBS. LLO or EqtII were then applied to the apical compartment and TEER was measured for 3 min. To measure effects of calcium influx, different concentrations of ionomycin calcium salt were added to the apical compartment and TEER was measured for 3 min. To determine the effect of potassium efflux we first washed Caco-2 cells with DMEM, then added different concentrations of nigericin to the apical compartment and measured TEER for 3 minutes.

### MTT and LDH cell viability assays

100 μl of Caco-2 cell suspension at a density 57 000 cells/ml were seeded per well on 96-well microtiter plates and cultured for 1 week in the test medium. For MTT assay, cells were washed with DMEM, treated with increasing concentrations of LLO or EqtII and incubated at 37°C for 3 hours. Cells were then washed with the test medium and incubated overnight. MTT cell viability reagent (Sigma-Aldrich) was added to the cells followed by 2 hour incubation at 37°C. The formed formazan crystals were dissolved in DMSO and absorbance was measured at 570 nm with Fluostar Galaxy microplate reader.

For LDH cell viability assay, identically cultured cells on 96-well microtiter plates were rinsed with DMEM, treated with increasing concentrations of LLO or EqtII and incubated at 37°C for 3 hours. After incubation, 90 μl of supernatant was transferred to another 96-well microtiter plate and centrifuged at 250 *g* for 4 min. 30 μl of supernatant was again transferred to a fresh microtiter plate, 70 μl of the LDH substrate (Promega) was added and absorption was measured at 490 nm after 15 min of incubation.

### SYTOX Green staining

100 μl of Caco-2 cell suspension at a density 57 000 cells/ml were seeded per well on 96-well microtiter plates and cultured for 1 week in the test medium. Cells were rinsed with DMEM and 50 μl of 30 μM SYTOX Green Nucleic Acid Stain (Molecular probes) was added, followed by addition of LLO or EqtII. Increase in fluorescence was measured at 520 nm with Fluostar Galaxy microplate reader.

### Occludin, claudin-1 and E-cadherin staining

Cells were washed with DMEM, treated with 125 nM LLO or 100 nM EqtII and incubated for 3–5 min. Untreated cells served as a negative control and cells treated with 2.5 mM EDTA for 30 min at 37°C as a positive control. Cells were washed with PBS (pH 7.4, 37°C) and fixed. Fixation for occludin staining was done with incubation in 95% ethanol for 30 min at 4°C followed by ice cold acetone for 3–5 min at room temperature. For claudin-1 staining 10 min fixation with a 1:1 (v:v) mixture of methanol and acetone was used. For E-cadherin staining cells were first fixed with 4% paraformaldehyde for 15 minutes at 37°C and then permeabilized with 0.1% Triton X-100 (v:v) for 15 minutes at 4°C. After fixation cells were washed 2 times with PBS and incubated for 30 min at room temperature with 2% (w:v) BSA solution. Mouse-anti-occludin, rabbit-anti-claudin-1 or mouse-anti-E-cadherin primary antibodies (Molecular probes) were added and incubated overnight at 4°C for occludin and claudin-1 or for 1 hour at room temperature for E-cadherin. After incubation, cells were washed 3 times with PBS for 10 min followed by incubation with goat-anti-mouse-Alexa488 or goat-anti-rabbit-Alexa488 secondary antibodies (Molecular probes). After 1 hour incubation at room temperature cells were washed 3 times with PBS for 5 min and imaged.

### Actin cytoskeleton staining

Cells were treated with 125 nM LLO or 100 nM EqtII and incubated for 3 to 5 min. Untreated cells served as negative control and cells treated with 2.5 mM EDTA for 30 min at 37°C as a positive control. Cells were then washed with PBS (pH 7.4, 37°C), fixed with 4% paraformaldehyde for 10 min and permeabilized with ice cold acetone for 3–5 min at room temperature. After washing the cells 3 times with PBS, they were incubated with 1% (w:v) BSA for 30 min and then Alexa fluor 488 phalloidin (Molecular probes) was added to the final concentration of 6 μM and incubated for 20 min at room temperature. Cells were washed again 3 times in PBS and 200 μl of 1 μg/ml DAPI was added. After a 5 min incubation cells were again washed 3 times in PBS and imaged.

### Confocal microscopy

100 μl of Caco-2 cell suspension at a density 178 000 cells/ml were seeded per well on chamber slides and cultured for 3 weeks in the test medium. Leica TCS SP5 laser scanning microscope mounted on a Leica DMI 6000 CS inverted microscope (Leica Microsystems, Germany) was used for imaging of stained cells. The inverted microscope was equipped with an HCX PL APO 63 × (NA 1.4) oil immersion objective lens. For sequential excitation, a 50-mW 405-nm diode and a 476-nm line of a 25-mW argon laser were used. The fluorescence emission was detected at 500 to 530 nm. Images were analyzed using Leica Application Suite advanced fluorescence lite (2.5.1 build 6757) program.

### Binding to multilamellar vesicles

Multilamellar vesicles (MLVs) were prepared by first mixing chloroform stock solutions of 1-palmitoyl-2-oleoyl-sn-glycero-3-phosphocholine (POPC) and cholesterol (1:1 mol:mol). Mixture was slowly dried under vacuum with rotary evaporator to obtain a uniform film on the surface of the flask. The flask was further dried under high vacuum for 1 hour. The lipids were resuspended in MLV buffer pH 5.7 (20 mM MES, 140 mM NaCl) with vortexing the mixture together with glass beads. The MLV suspension was transferred to a fresh tube and freeze-thawed in liquid nitrogen 3 times. The MLVs were stored at -20°C for up to 2 weeks. LLO^A318C-L334C^ was oxidized or reduced in the same way as for TEER experiments (see above). The entire procedure was carried out at room temperature. MLV suspension was added to mutant protein in molar ratio 3000:1 (lipid:protein). The mixture was incubated for 20 minutes and centrifuged for 15 minutes at 16 000 *g*. Supernatant was removed and fresh MLV buffer was added to resuspend the pellet. The MLVs were pelleted again and the second supernatant was discarded. The resulting pellets together with supernatants were used for the SDS-PAGE analysis.

### Hemolytic activity

LLO^A318C-L334C^ was oxidized or reduced in the same way as for TEER experiments (see above). Bovine red blood cells (RBCs) were washed three times with RBC buffer pH 7.4 (20 mM Tris, 140 mM NaCl) and diluted in the same buffer to yield A_630_ = 1. RBC suspension was added to a serial dilution of LLO^A318C-L334C^ and decrease in turbidity of RBC suspension was followed with Synergy 2 microplate reader (Biotek) for 20 minutes at 25°C. Maximal rate of turbidity decrease was determined for each well and plotted against protein concentration.

### Western blot

Cells were cultured for 3 weeks on transwell permeable supports. Prior to the experiment cells were washed in DMEM and then treated with 31.3 nM LLO, 25 nM EqtII or 2.5 mM EDTA. Negative controls were untreated cells. After 5, 30 or 60 minutes cells were washed with warm PBS, lysed with RIPA buffer for 20 minutes at 4°C and centrifuged for 10 minutes at 20 000 *g*. Supernatants of cell lysates were then subjected to SDS-PAGE and western blotting using rabbit-anti-claudin-1 primary antibodies and horseradish conjugated goat-anti-rabbit secondary antibodies.

### Permeability of Caco-2 monolayer

Caco-2 cells were cultured on transwell permeable supports for 3 weeks. Prior to the experiment they were washed in DMEM and treated with different concentrations of LLO or EqtII. EDTA treated (2.5 mM final concentration) and untreated cells were used as a positive and negative control, respectively. Prior to the treatment, FITC-dextran 3 or fluorescein were added to the apical compartment to the final concentration of 0.2 or 0.01 mg/ml, respectively. After 30, 60, 120 and 180 min 100 μl of medium was taken from the basal compartment and substituted with the same amount of DMEM. Samples were collected on a 96-well microtiter plate. Their fluorescence was measured at 520 nM with Fluostar Galaxy microplate reader and apparent permeability coefficient (P_app_) was calculated using the following equation:

$$Papp\;=\;\frac{dQ}{dt*A*Co}$$

where dQ/dt is the permeability rate, *A* is the diffusion area of the monolayer, and C_0_ is the initial concentration of the tested substance in the apical compartment.

## Results and Discussion

### LLO causes a drop in TEER that is irreversible at high toxin concentrations

**Fig 1:**
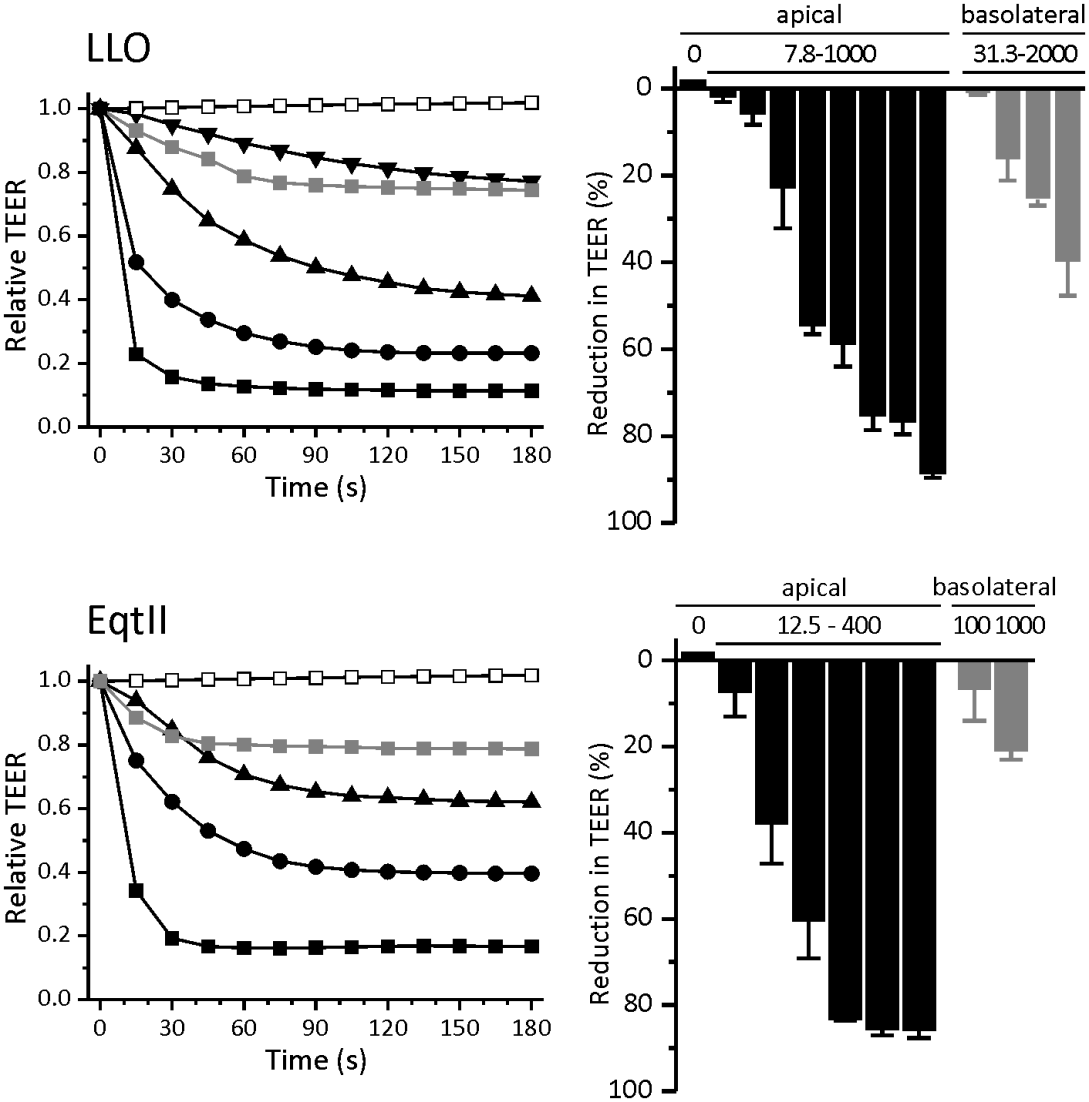
Effect of LLO and EqtII on TEER. Top left: Time course of the relative drop in TEER after apical application of 1 μM (black squares), 250 nM (black circles), 62.5 nM (black triangles) and 15.6 nM (black inverted triangles) or basolateral application of 2 μM (gray squares) LLO and control (white squares). Bottom left: Time course of the drop in TEER after apical application of 100 nM (black squares), 50 nM (black circles) and 25 nM (black triangles) or basolateral application of 1 μM (gray squares) EqtII and control (white circles). Top right: Relative drop in TEER 3 minutes after apical (black; at two-fold increase in concentration from 7.8 nM-1 μM) or basolateral (gray; a four-fold increase from 31.3 nM-2 μM) application of LLO. Bottom right: Relative drop in TEER 3 minutes after apical (black; at two-fold increase in concentration from 12.5–400 nM) or basolateral (gray) application of EqtII. Data represent means of percent of initial values. Error bars are the standard error of the mean calculated for 2 to 4 independent experiments.

In our current study we aimed to examine the effects of LLO pore-formation on the intestinal epithelium model cell line Caco-2 and compare them to effects of another non-related pore-forming toxin EqtII. First we measured the impact of the toxin on TEER values. Namely, a drop in TEER value is a very sensitive indicator of the barrier disruption. For example, membrane perturbations by saponines have been shown to cause a drop in TEER and cytoskeletal changes that result in disruption of tight junction proteins and subsequent increase in paracellular permeability [54]. TEER values of Caco-2 cells used in our experiments ranged from 2 to 3 kΩ*cm^2^, which indicated that Caco-2 cell monolayer was mature and intact [42]. Caco-2 cells were treated with increasing concentrations of LLO. The toxin was added to the apical compartment and a dose dependent drop in TEER was observed (Fig 1). At the highest concentration of LLO applied apically (1 μM) TEER value dropped by more than 80% of the initial value in the first 30 seconds. At lower concentrations of LLO the drop was slower and less pronounced. Apical application of 125 nM LLO caused a 60% drop in TEER after 3 minutes, whereas 100 nM EqtII resulted in a drop of more than 80% of initial value. Furthermore, apical application of LLO yielded a much slower response than EqtII and concentrations of LLO needed to achieve the same short term effect as EqtII were about 10 times higher (Fig 1).

Due to polarization of Caco-2 cells, proteins and lipids are differentially distributed on the apical and basolateral side. Therefore we compared the apical application of LLO to the basolateral one. Fig 1 shows that basolateral addition of 2 μM LLO causes a drop of only down to 40% of the initial value, which is notably lower than 1 μM LLO added from the apical side. Similar results were obtained for EqtII, where the apical application of EqtII caused a dose-dependent drop in TEER with maximum effect at a 100 nM concentration (Fig 1). The drop in TEER when EqtII was added from the basolateral side at 1 μM concentration caused a drop of only about 20% of the initial value. The reason for the differential response may be the lipid receptor distribution and accessibility, because of the distinct protein and lipid composition of the apical and basolateral side, which can affect binding of toxins and their effects on the monolayer [55,56]. EqtII specifically binds to sphingomyelin and its uneven distribution in the cell membrane could explain why EqtII added from the apical side elicits a greater response than when it’s added from the basolateral side [57]. There is a possibility that the permeable support on which the cells are grown provides only a limited accessibility of the basolateral membrane. However, this may not be the main issue since lysenin, the pore-forming protein from the earthworm *Eisenia foetida*, labels MDCK cells mostly when added to cells from the basolateral side [58].

**Fig 2:**
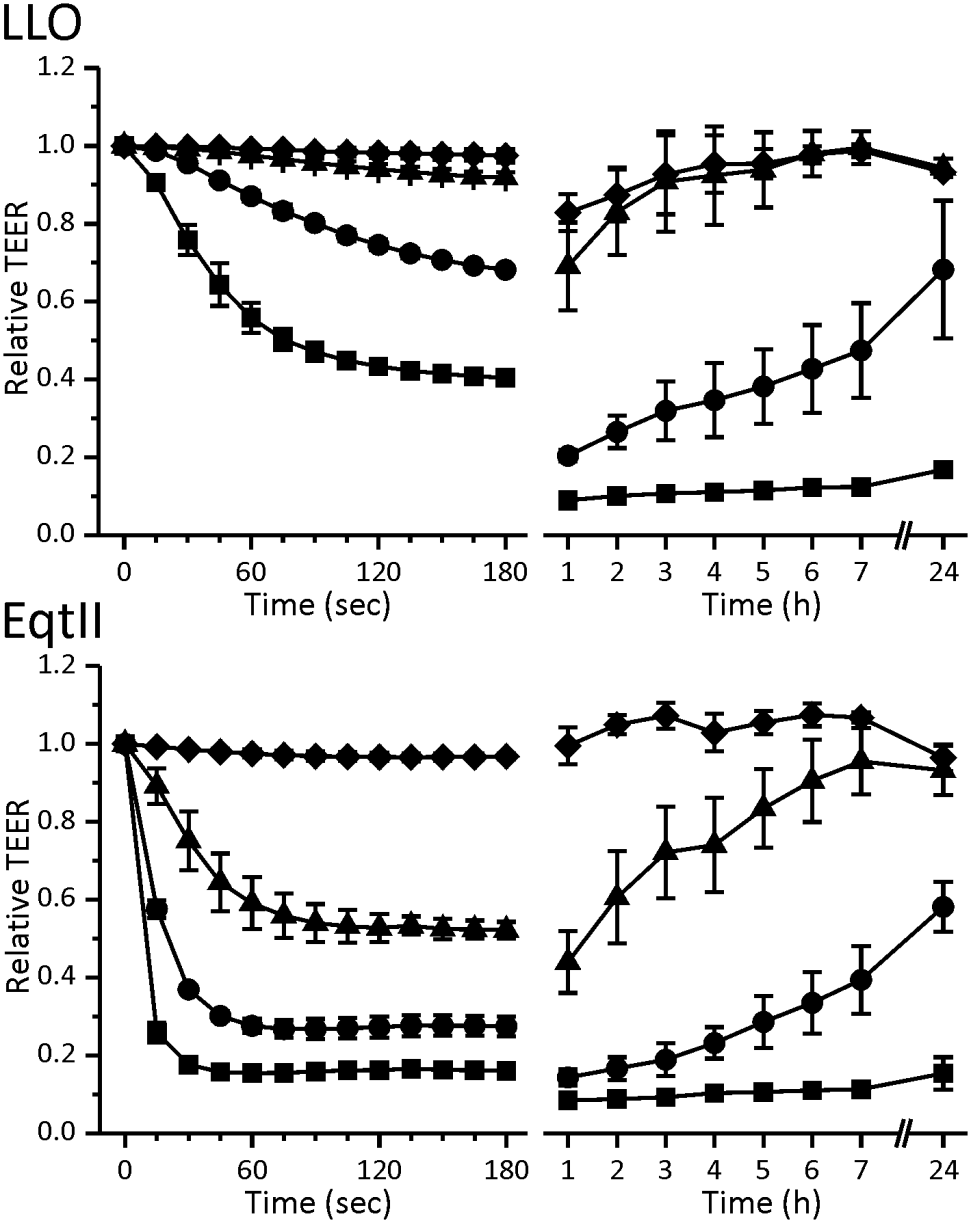
Regeneration of the Caco-2 monolayer after apical treatment with LLO and EqtII. Top: Time course of relative TEER values after apical application of 62.5 nM (squares), 31.3 nM (circles), 15.6 nM (triangles) and 7.8 nM (diamonds) LLO. Bottom: Time course of relative TEER values after apical application of 100 nM (squares), 50 nM (circles), 25 nM (triangles) and 12.5 nM (diamonds) EqtII. Data represent means of percent of initial values. Error bars are the standard error of the mean calculated for 2 to 3 independent experiments.

Since not every attack of pore-forming toxins results in cell death we wanted to see if Caco-2 cell monolayer as a whole is able to regenerate its TEER values after treatment with LLO and EqtII and, if so, how long does this process take. The time needed for regeneration was dose dependent (Fig 2). Complete regeneration of TEER values and thus the monolayer was observed only for the lower effective concentrations, i.e. 15.6 nM for LLO and 25 nM for EqtII, respectively. Although the fast drop in TEER reached its plateau after 3 minutes, the effect continued at a slow rate and 1 hour after the treatment the lowest values were measured (Fig 2). A dose dependent slow increase in TEER followed after that. The decrease in TEER one hour after toxin treatment was 30–40% for 31.3 nM LLO and was less pronounced than for EqtII, in agreement with slower TEER response observed for LLO. Similar TEER measurements in the presence of LLO were reported for HT-29/B6 cells, where it was established that apical addition of LLO to cells caused a significant drop in TEER that was not completely reversible at higher toxin concentration [32]. A dose-dependent drop in TEER of a monolayer was also measured for two other CDCs, anthrolysin O (ALO) from *Bacillus anthracis* on Caco-2 cell line derived clone C2BBE [59,60] and pneumolysin (PLY) from *Streptococcus pneumonia* on BBMEC (bovine brain microvascular endothelial cells) [61]. A drop in TEER is an indicator of barrier disruption, which diminishes the epithelium’s capacity to prevent uncontrolled passage of molecules. This presents an opportunity for pathogenic bacteria to cross the monolayer and invade the underlying tissues.

**Fig 3:**
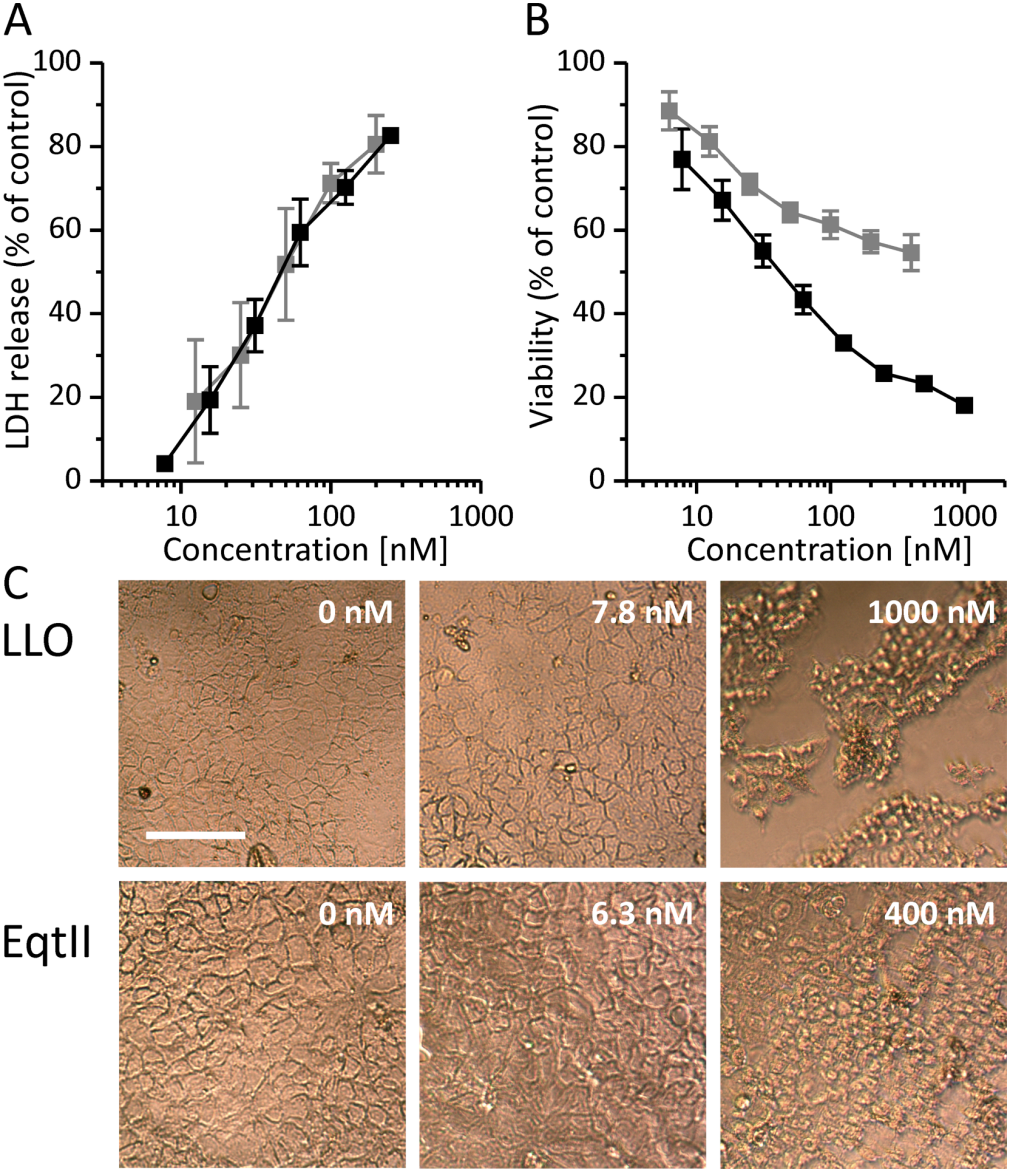
Cell viability after treatment with LLO and EqtII. **A** Relative LDH release 3 hours after treatment with LLO (black) or EqtII (gray). **B** MTT cell viability assay. Relative cell viability 24 hours after treatment with LLO (black) or EqtII (gray). **C** Light microscopy of Caco-2 cells 24 hours after treatment with LLO (top) or EqtII (bottom). Data represent means of percent of negative controls. Error bars are the standard error of the mean calculated for 2 to 3 independent experiments. Bar in panel C represents 100 μm.

In order to correlate the drop in TEER after exposure to LLO or EqtII with their effects on viability of Caco-2 cells we performed LDH and MTT assays. We measured LDH release from ruptured cells 3 hours after the treatment (Fig 3A) while the viability after 24 hours was derived from MTT signal in cells that were still viable (Fig 3B). LDH leakage from the cells was dose-dependent and very similar for both toxins at identical molar concentrations, suggesting comparable dying rate. On the other hand 24 hours after treatment MTT signal of viable cells was clearly higher in EqtII treated cells. Even at the highest concentrations of EqtII used (400 nM), the MTT signal was above 50% of control value, whereas treatment with comparable concentration of LLO (500 nM) resulted in less than 25% of viable cells. TEER recovery profiles (Fig 2) indicate that surviving Caco-2 cells recover faster after EqtII addition when compared to LLO. The low viability signal 24 hours after LLO treatment could also be explained by cells entering a quiescent-like state which has previously been reported for LLO [27]. MTT cell viability assay is based on metabolically active cells reducing the tetrazolium dye MTT to its insoluble form formazan. Cell number could thus be underestimated in MTT assay, if they entered into the low metabolic state. Gonzales *et al*. compared the recovery of HT29 cells after treatment with LLO and aerolysin [27]. Aerolysin is a pore-forming toxin from *Aeromonas hydrophila*, which forms pores of a size similar to EqtII [27]. In contrast to our results, the recovery of cells after treatment was slower for the small aerolysin pores and much faster for large LLO pores. EqtII has also been shown to inhibit endocytosis [62], which is one of the major pathways of membrane repair after attack by pore-forming proteins [16,63–65].

### Drop in TEER is a result of pore formation and disruption of a tight junction protein claudin-1

**Fig 4:**
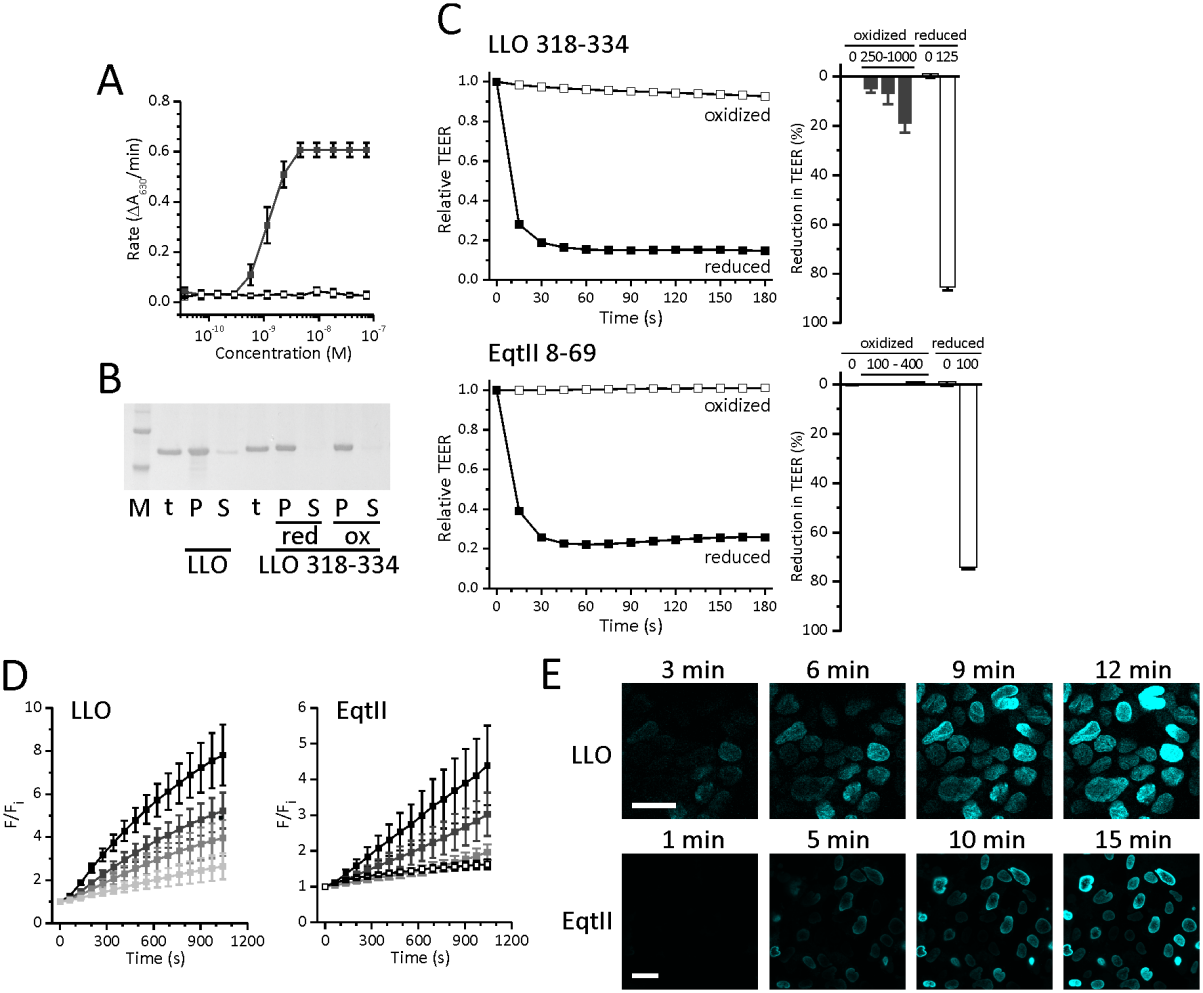
Pore formation with LLO or EqtII in Caco-2 cells. **A** Hemolytic activity of LLO^A318C-L334C^ in the reduced state (filled symbols) or the oxidized state (open symbols). Average ± S.D, n = 5. **B** Binding of LLO and LLO^A318C-L334C^ to multilamellar vesicles. M, molecular weight markers (the bottom band is 50 kDa and the upper is 60 kDa). t, total applied protein; P, protein associated with the pelleted fraction; S, protein remaining in the supernatant after centrifugation. **C** Time course of the relative drop in TEER (left) after apical application of oxidized (white) or reduced (black) LLO^A318C-L334C^ or EqtII^V8C-K69C^ and a relative drop in TEER (right) 3 minutes after apical application of oxidized (dark gray) or reduced (white) LLO^A318C-L334C^ or EqtII^V8C-K69C^. Data represent means of percent of initial values. Error bars are the standard error of the mean calculated for 2 to 3 independent, experiments. **D** Time course of SYTOX Green staining after application of 125 nM (black), 62.5 nM (dark gray), 31.3 nM (gray) and 15.6 nM (light gray) LLO (left) or 100 nM (black), 50 nM (dark gray) and 25 nM (gray) EqtII (rigt) and control (white squares). **E** Confocal microscopy of SYTOX Green staining after application of 125 nM LLO or 100 nM EqtII. Bar in panel C represents 30 μM.

To show that formation of pores at plasma membranes results in a drop in TEER we applied double cysteine mutants, LLO^A318C-L334C^ and EqtII^V8C-K69C^. Pore formation by proteins is commonly associated with significant conformational rearrangements that allow insertion of the protein in the membrane and formation of the final pore. These rearrangements can be blocked by introducing two cysteine residues at particular positions, so that upon formation of the disulfide bond the protein is locked in a certain position and pore formation cannot be complete. In order to block permeabilizing activity of LLO we introduced cysteine residues to two helices of the transmembrane helix 2 region, A318C and L334C, which during pore formation rearranges and forms one of the transmembrane β-hairpins [66]. This double mutant did not exhibit hemolytic activity in the oxidized state, but was hemolytic when protein was reduced prior to our measurements (Fig 4A). We have also verified that the LLO^A318C-L334C^ bound to the lipid membranes to the same extent in reduced or oxidized state (Fig 4B). When 1 μM LLO^A318C-L334C^ was applied under oxidative conditions, the drop in TEER was about 20% of the initial value. However, when 125 nM LLO^A318C-L334C^ was applied under reducing conditions the drop in TEER was over 80% (Fig 4C). We have previously characterized EqtII^V8C-K69C^ mutant, which prevents dislocation of the N-terminal region of EqtII, which is crucial for pore formation. This mutant can bind to membranes in a similar manner as the wild-type protein, but it cannot form pores [53,67]. Under reducing conditions, 100 nM EqtII^V8C-K69C^ caused over 70% drop in TEER, but the mutant did not affect TEER in oxidative conditions, even if it was applied at four times higher concentration (Fig 4C). Results on mutant proteins indicate that the drop in TEER is in large a consequence of pore formation and not toxin binding to the cell membrane, since TEER values drop significantly only when a functional pore is formed.

We also assessed pore formation by nucleic acid staining with a cell non-permeant dye SYTOX Green. Caco-2 cells were treated with LLO or EqtII after the addition of SYTOX Green and staining was followed by microtiter plate reader or confocal microscope (Fig 4D and 4E). Since this dye does not cross intact membranes, staining of the cell interior with SYTOX Green indicates that pores had to be formed on the plasma membrane in order to allow the entry of the dye into the cells. The staining intensity was time and toxin concentration dependent (Fig 4D and 4E). It appeared in almost all cells even at low toxin concentrations. Furthermore, the staining didn’t coincide with any morphological changes making necrotic membrane rupture an unlikely reason for staining. These results altogether clearly indicate that the drop in TEER values correlates in large part with the formation of pores in the plasma membrane.

**Fig 5:**
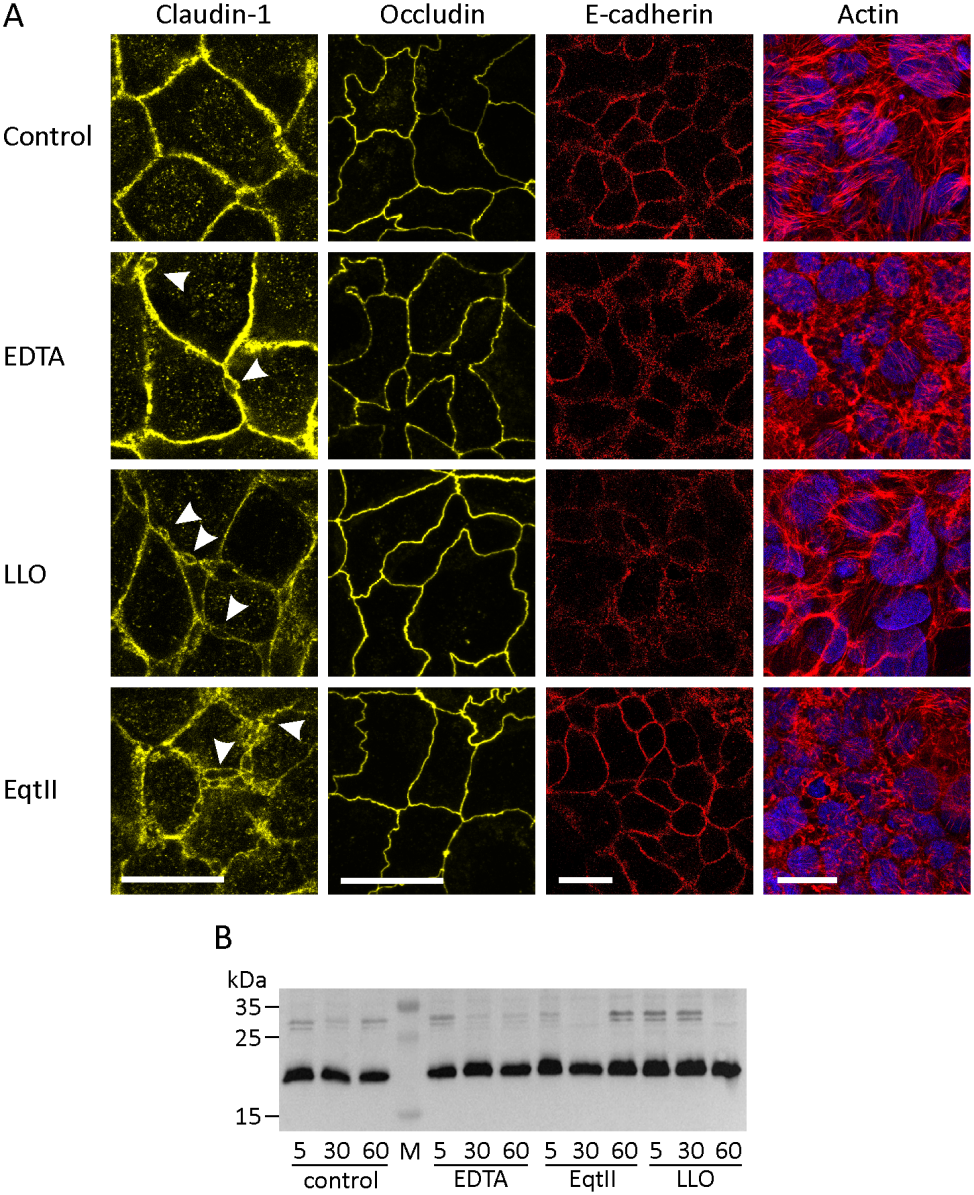
Effects of LLO and EqtII on tight junction proteins and actin. **A** Immunostaining and confocal microscopy of claudin-1 (first column), occludin (second column), E-cadherin (third column) and actin and nuclei (fourth column) after treatment with LLO and EqtII. Arrows indicate ruptures of tight junctions. Scale bars represent 20 μm. **B** Western blot analysis of claudin-1 after 5, 30 and 60 minute exposure to 31.3 nM LLO, 25 nM EqtII, 2.5 mM EDTA and untreated cells (control). M, molecular weight marker.

The primary function of tight junctions in intestinal epithelium is to restrict free passage of molecules and ions between neighboring enterocytes. This permeability control is further supported by adherens junctions, mainly by maintaining the tissue structure. Barrier function impairment by intestinal pathogens is often caused through epithelial lesions or changes in the structure of epithelial tight junctions [68]. Barrier properties of tight junctions are defined by claudins and occludin [69–71]. Claudins are also linked to F-actin, which can affect sorting of tight junction proteins and thus the barrier function [72]. Therefore, we followed the effect of LLO and EqtII on two tight-junction proteins that play important roles in the tight-junction structure, claudin-1 and occludin, as well as the actin cytoskeleton and adherens junction protein E-cadherin (Fig 5A). Because calcium ions have been shown before to affect organization of junctional proteins and thus paracellular permeability [73,74], we used 2.5 mM EDTA as a positive control to demonstrate rearranged tight junction structure. LLO showed a clear effect on claudin-1, E-cadherin and actin arrangement, but not on occludin (Fig 5A). The latter was affected only by chelation of extracellular calcium with EDTA. EqtII affected claudin-1 and actin but not occludin or E-cadherin. Rearrangement of claudin-1 structure was much more pronounced when Caco-2 cells were treated with LLO or EqtII than when they were treated with EDTA (Fig 5A). Despite the noted effect on claudin-1 by these toxins, no protein degradation could be shown (Fig 5B). The effect of a pore-forming toxin on tight junction proteins was also shown for aerolysin on HT-29/B6 cells where they observed opening of tight junctions, redistribution of claudin-1, -4 and -5, occludin and zonula occludens 1 [75]. Our results show that the action of both toxins on the apical membrane leads to a similar profound effect on claudin-1 distribution and on the organization of the actin cytoskeleton. Seeing these two effects in unison is to be expected since the actin cytoskeleton and tight junction proteins are connected [76]. Our results also show that both LLO and EqtII not only affect the cell membrane and thus transcellular permeability, but also the structure of tight junctions, which may lead to increased paracellular permeability to ions and the resulting drop in TEER. Although these toxins form pores on the cell membrane, it is not likely that the drop in TEER is mainly a result of transcellular ion fluxes through these pores. Caco-2 cells are polarized with tight junctions separating the apical and basolateral membrane. Thus the pores are most likely formed only on the apical and not also on the basolateral membrane which prevents the direct transcellular passage of ions. However, pore formation causes ion fluxes along concentration gradients, which disrupts the ion balance inside the cell. The cell may in turn react by activating ion channels to compensate for any loss or excess of ions. By actively pumping ions across the membranes the cell may also affect transcellular ion currents, which reflects in changed TEER.

**Fig 6:**
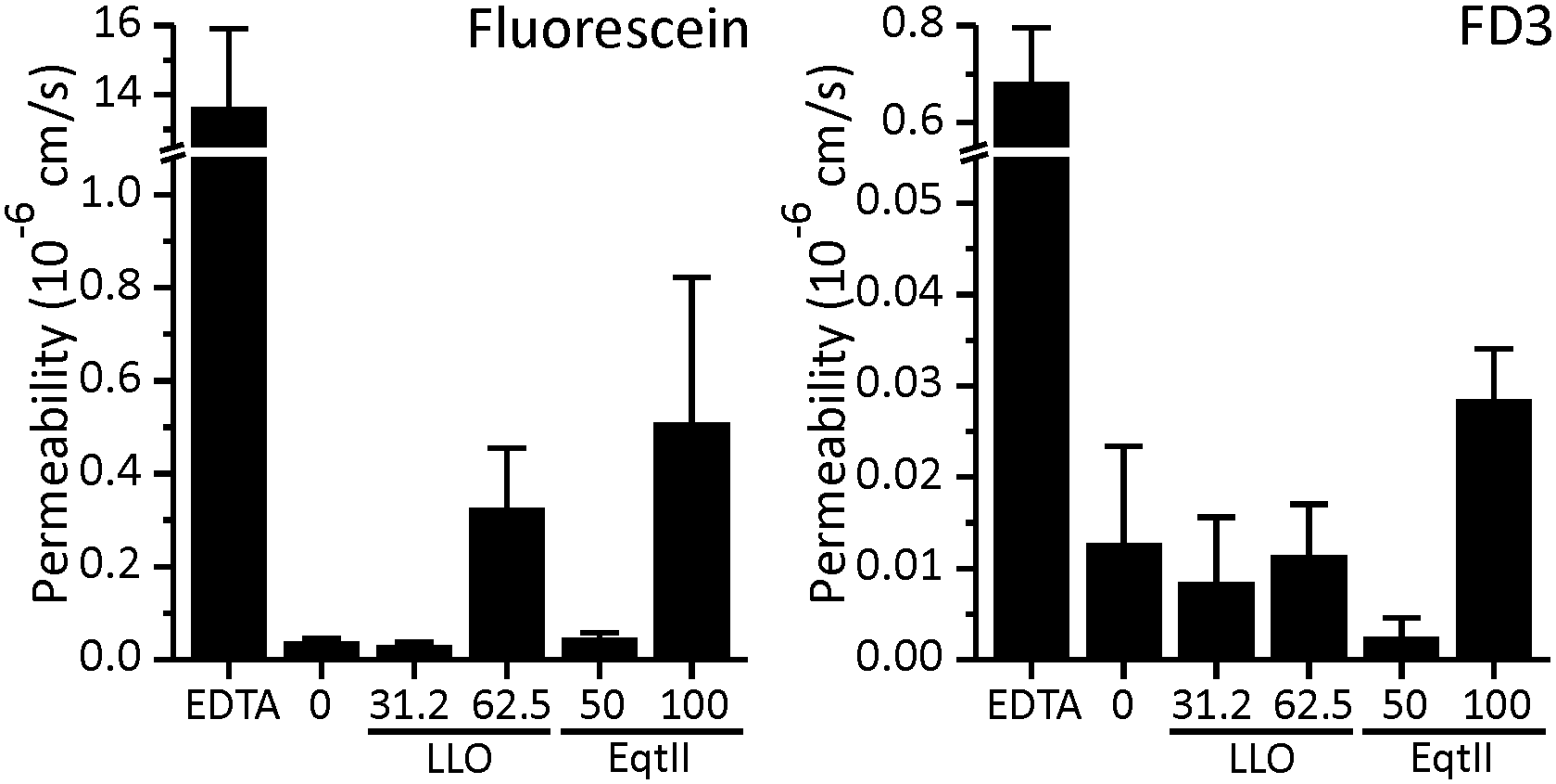
Effects of LLO and EqtII on permeability of Caco-2 cell monolayer. Permeability of Caco-2 monolayer to fluorescein and FD3 after treatment with LLO and EqtII. Data represent means of permeability coefficients ± standard error for 2 to 3 independent experiments.

Tight junction structures contribute to TEER by creating a barrier for paracellular passage of ions and other solutes [77]. Since we noted the effect of LLO and EqtII on these structures, we wanted to see if this correlates with an increase of paracellular permeability to certain molecules. Also, Caco-2 cell monolayers have been widely used to assess the transepithelial movements of various substances like drugs, amino acids and metal ions [40–42,78,79]. If LLO or EqtII could accelerate the passage of certain molecules across the intestinal barrier without compromising the integrity of the epithelium, they could potentially be used to improve absorption of orally administered drugs in the intestinal tract. We used fluorescently labeled 3 kDa Dextran (FD3) and fluorescein (M_w_ = 332 Da) as examples of large and small molecules, respectively, to establish the size range of molecules that can pass through the monolayer after the exposure to toxins (Fig 6). EDTA at 2.5 mM concentration was used as a positive control. We observed higher permeability of both test molecules in the presence of EDTA (Fig 6). However, the addition of LLO and EqtII to Caco-2 cell monolayer resulted in a negligible increase in permeability of FD3 or fluorescein (Fig 6). These results indicate that paracellular permeability may be increased only for ions, but not for any larger molecules. Similar to our results, two other members of the CDC family have also been shown to affect certain tight junction proteins and the actin cytoskeleton but in a very distinct manner. ALO has been shown to affect occludin and increase the permeability of Caco-2 monolayer to 3 kDa FITC-dextran, while LLO did not. ALO also did not affect E-cadherin and the actin cytoskeleton [59,60]. On the other hand, PLY had a specific effect on the actin cytoskeleton. It caused actin polymerization and formation of stress fibers, lamelipodia and filopodia [80,81], whereas our results show that treatment with LLO leads to disruption of the cytoskeleton structure. Tight junction proteins and the associated cytoskeleton seem to be a common target for pore-forming proteins such as CDCs, although the affected protein within the tight junction complex and the effect on actin seems to differ between different CDCs.

### Calcium influx affects magnitude of the response

**Fig 7:**
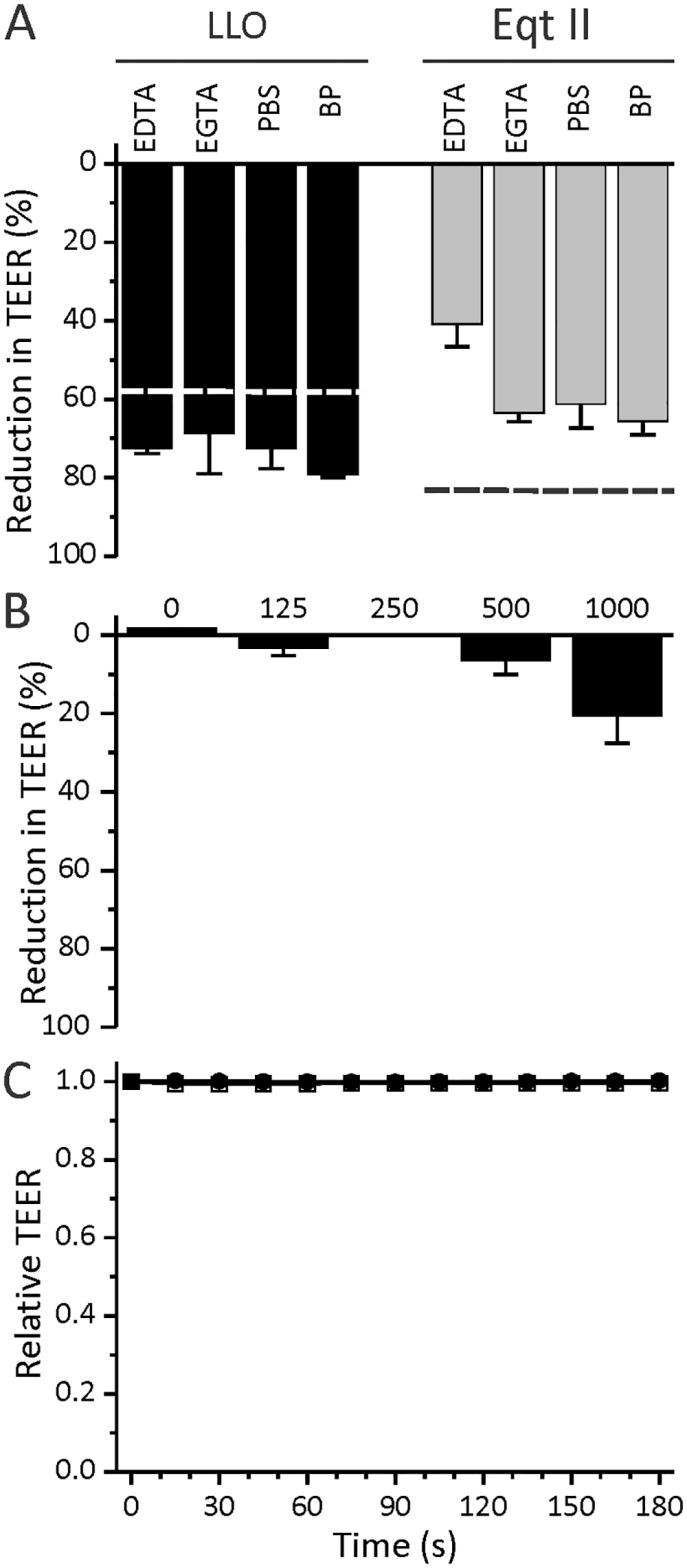
Effects of calcium on TEER drop. **A** Effects of intra- and extracellular calcium chelation on the action of 125 nM LLO and 100 nM EqtII. For extracellular chelation 2.5 mM EDTA or EGTA was used or the medium was substituted by PBS. For intra- and extracellular chelation BAPTA-AM and PBS (BP) were used. Values for control experiments in DMEM (from Fig 1) are shown by the dashed line for each protein. **B** Effects of different concentrations (nM) of ionomycin on TEER. **C** Time course of relative TEER values after apical application of 4 μM nigericin (filled symbols) and control (open symbols).

Formation of toxin pores in membrane results in passage of ions and other small molecules through them along their concentration gradients [82,83]. Calcium ions are well known signaling molecules. Influx of calcium through toxin pores has been shown to induce membrane repair with endocytosis, activate NF-κB and IL-8 production, phosphorylation of MAP kinase p38, cause stress of the endoplasmatic reticulum and potentiate entry of bacteria into host cells [15,16,33,63,64,84]. The intracytosolic concentration of calcium ions can increase either by their influx via toxin pores along their concentration gradient or by their release from intracellular stores [32,85]. We, therefore, assayed the role of calcium during the pore forming toxins induced reduction in TEER. We removed calcium from the external medium by either chelation with 2.5 mM EDTA or EGTA, or simply by replacing the medium (DMEM) with PBS (Fig 7A). Since it was shown that calcium can be released from the intracellular stores as a result of LLO treatment [32], we also chelated both extra- and intracellular calcium. This was done by loading the cells with 50 μM BAPTA-AM prior to the experiment along with replacing the medium (DMEM) with PBS. Once inside the cell, BAPTA-AM would chelate the calcium that was already present in the cytosol as well as the calcium that would potentially be released from intracellular stores like the endoplasmic reticulum. Removing calcium from the external medium had a negligible effect on the drop in TEER caused by LLO. On the other hand, the action of EqtII on Caco-2 monolayer was much more sensitive to the effects of calcium chelation. The drop in TEER after addition of 100 nM EqtII was reduced upon chelation and the effect was most pronounced with EDTA, which is less specific for calcium ions and also readily binds other divalent cations. Taken together these data show that calcium plays a role in regulation of epithelial response to pore forming toxins, however it doesn’t seem to be the key regulator of the process. To additionally show that calcium influx is not the sole cause of the drop in TEER, we treated the cells with calcium specific ionophore ionomycin (Fig 7B). Different concentrations of ionomycin were added to the apical compartment and TEER was measured for 3 min. 1 μM ionomycin caused only a 20% drop in TEER, whereas the same concentration of LLO resulted in a drop of over 90% of the initial value (Fig 1).

Potassium eflux through toxin pores has been shown to induce inflammasome activation, histone modifications, activation of certain MAP kinases, activation of autophagy, arrest in protein synthesis and lipid droplet formation [27,86]. Therefore, we additionally examined the effect of nigericin, a potassium specific ionophore on the Caco-2 monolayer (Fig 7C). We added different concentrations of nigericin to the apical compartment and measured TEER for 3 minutes. Contrary to LLO and ionomycin, nigericin did not elicit a drop in TEER even at 4 μM concentration. The results clearly show that calcium contributes to only a small portion of TEER drop, and is not the sole cause of it. This further indicates that more than one ion or molecule is involved in this phenomenon, supporting our hypothesis that pore formation is the main cause of TEER decrease upon toxin activity towards cells.

## Conclusions

In the current study we show that despite the difference in evolutionary origin, lipid specificity and pore forming mechanism between LLO and EqtII, both toxins elicit similar effects on Caco-2 cell monolayer. They both caused a significant dose-dependent drop in TEER, which was more pronounced when the toxins were added from the apical side. However, apical application of LLO yielded slower response than EqtII and higher concentrations of LLO were needed to achieve the same effect. The drop in TEER was due to pore-formation and associated changes in actin cytoskeleton and tight junctions, but independent of calcium. Our results indicate that LLO can efficiently compromise enterocyte barrier by acting upon their apical membrane. We show that the effect can accumulate over time and even small concentrations of LLO result in compromised epithelium integrity. Treatment with small concentrations of LLO resulted in slow but significant increase of LDH activity in the medium suggesting dying of some cells within the epithelium and could be relevant *in vivo* for a potential colonization of submucosa by bacteria.
